# Supplementary material for: Ligand-Coupled Conformational Changes in a Cyclic Nucleotide-Gated Ion Channel Revealed by Time-Resolved Transition Metal Ion FRET
Source: bioRxiv. 2024 Oct 2:2024.04.25.591185. Preprint. [Version 2] doi: 10.1101/2024.04.25.591185 (PMC11475872; doi:10.1101/2024.04.25.591185)
Supplement: Supplement 1 [file NIHPP2024.04.25.591185v2-supplement-1.pdf]

bioRxiv preprint doi: https://doi.org/10.1101/2024.04.25.591185; this version posted October 2, 2024. The copyright holder for this preprint (which was not certified by peer review) is the author/funder, who has granted bioRxiv a license to display the preprint in perpetuity. It is made available under aCC-BY 4.0 International license.

Supplemental Figures

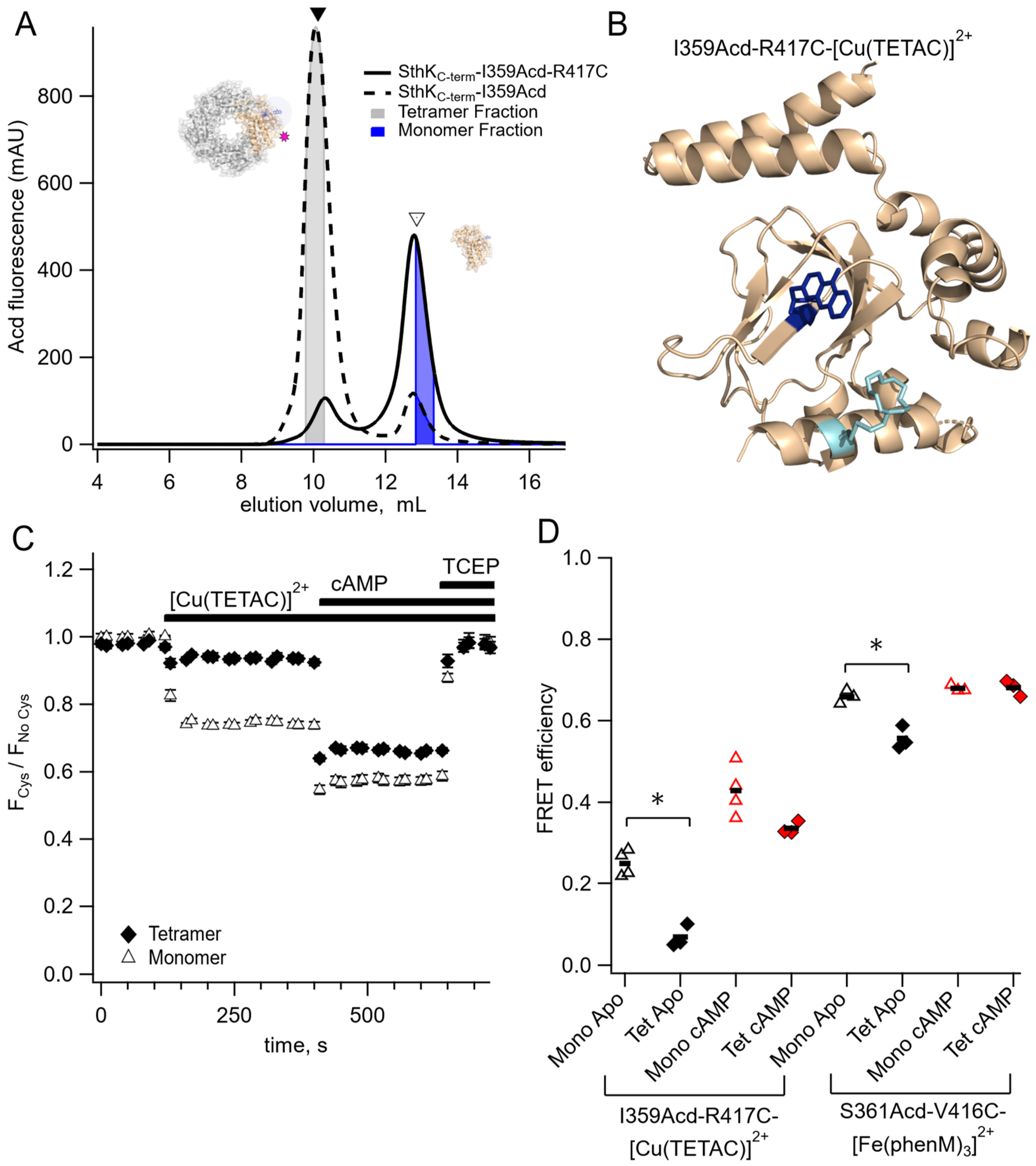

Fig. S1. Steady-state tmFRET of monomeric and tetrameric $SthK_{C\text{-term}}$. **(A)** SEC chromatogram showing separation of monomeric (empty triangle, blue area) and tetrameric protein (solid triangle, gray area) fractions for $SthK_{C\text{-term}}$-I359Acd and $SthK_{C\text{-term}}$-I359Acd-R417C (no WT $SthK_{C\text{-term}}$ present) on a Superdex 75 increase 10/300 column (GE Healthcare). **(B)** Structure showing $SthK_{C\text{-term}}$-I359Acd with $[Cu(TETAC)]^{2+}$ acceptor at R417C (PDB:4D7T). **(C)** Fluorescence time-course of $SthK_{C\text{-term}}$-I359Acd-417C upon addition of $[Cu(TETAC)]^{2+}$, cAMP and TCEP in tetrameric protein (closed diamonds) and monomeric protein (open triangles). **(D)** Summary of FRET efficiencies for monomer and tetramer, with mean values shown as horizontal lines. Left: $SthK_{C\text{-term}}$-I359Acd-R417C-$[Cu(TETAC)]^{2+}$ (apo, *P=0.0005, cAMP, P=0.06). Right: $SthK_{C\text{-term}}$-S361Acd-V416C-$[Fe(phenM)_3]^{2+}$ apo, *P=0.006, cAMP: P=0.9).

bioRxiv preprint doi: https://doi.org/10.1101/2024.04.25.591185; this version posted October 2, 2024. The copyright holder for this preprint (which was not certified by peer review) is the author/funder, who has granted bioRxiv a license to display the preprint in perpetuity. It is made available under aCC-BY 4.0 International license.

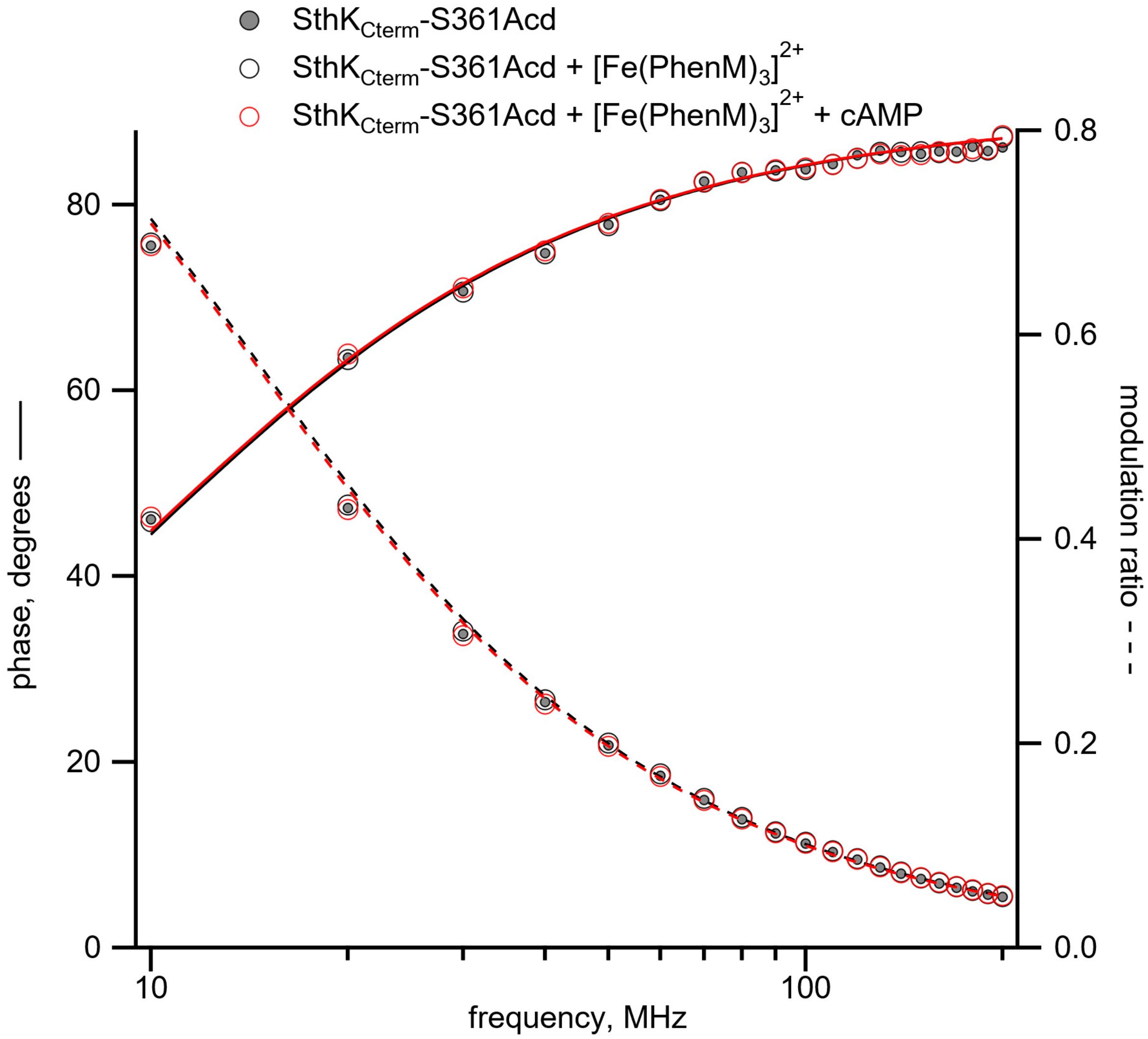

Fig. S2. Representative Weber plot of measured lifetimes of SthK$_{C\text{-}term}$-S361Acd donor-only (grey), then in the presence of $[Fe(phenM)_3]^{2+}$ (black), and after the addition of 1.23 mM cAMP (red), showing no change in lifetimes in the absence of a cysteine residue.

bioRxiv preprint doi: https://doi.org/10.1101/2024.04.25.591185; this version posted October 2, 2024. The copyright holder for this preprint (which was not certified by peer review) is the author/funder, who has granted bioRxiv a license to display the preprint in perpetuity. It is made available under aCC-BY 4.0 International license.

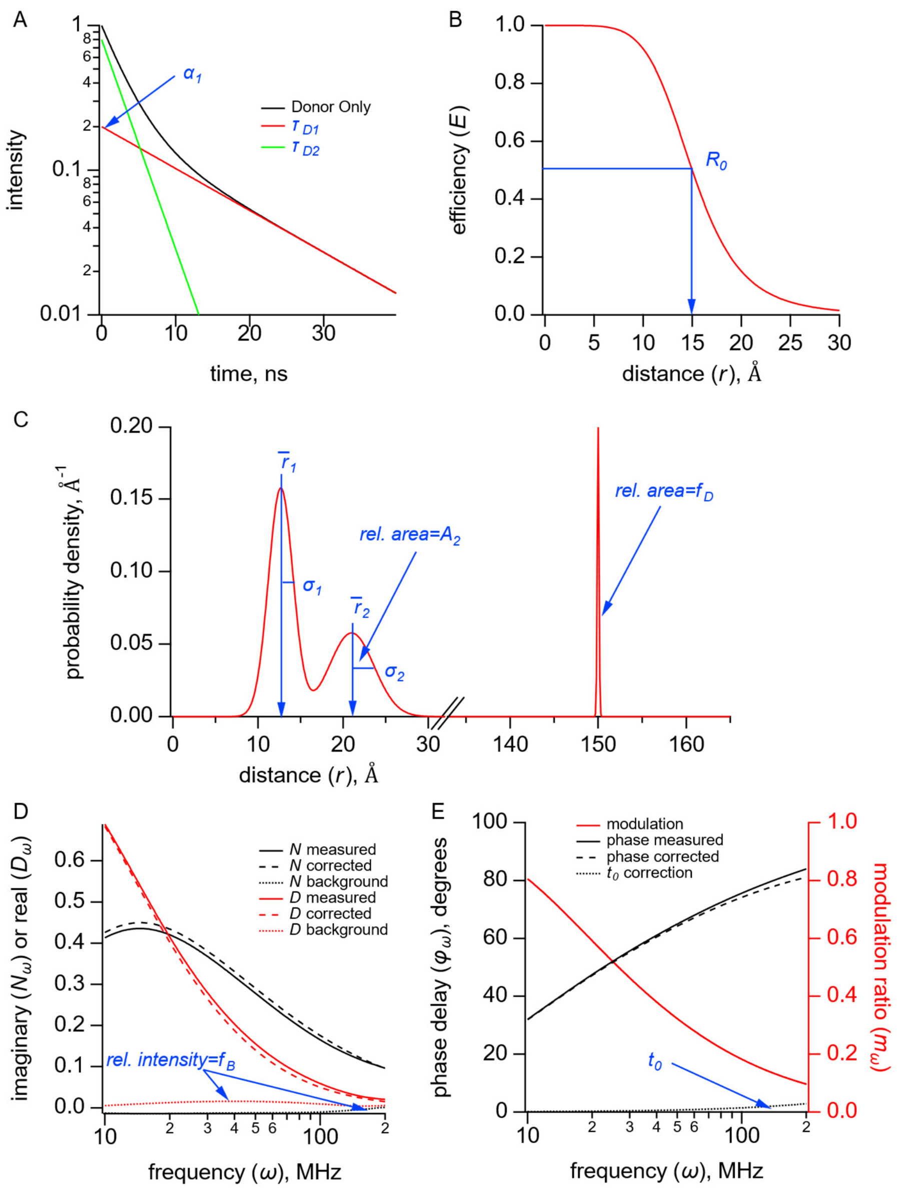

Fig. S3. Parameters used in the lifetimes fitting model for both the single and sum of two Gaussian approaches, shown in blue. **(A)** Graph of donor-only fluorescence-lifetime decay with two exponential components with time constants (τ_{D1}, τ_{D2}) **(B)** FRET efficiency (*E*) plot as a function of distance (*r*) between donor and acceptor and the R_0 values for 50% FRET transfer. **(C)** Probability distribution plot of donor and acceptor distances P(*r*) showing the sum of two Gaussian distributions, each with their own average distance ($\overline{r_1}$ and $\overline{r_2}$), standard deviations (σ_1 and σ_2) and relative amplitude of the second component (A_2). The donor-only fraction (f_D) is modeled as a narrow Gaussian with mean distance of 150 Å and standard deviation of 0.1 Å, beyond a detectable amount of FRET. **(D)** The in-phase (D_ω) and out-of-phase (N_ω) components are plotted for the measured, corrected, and background fluorescence signal as a function of the excitation modulation frequency (ω), where f_B is the fraction of the fluorescence intensity due to the background. **(E)** Plot of the phase delay (φ_ω) and modulation ratio (m_ω) of the measured and corrected fluorescence response as a function of the modulation frequency (ω) where t_0 is the time shift of the instrument response function.

bioRxiv preprint doi: https://doi.org/10.1101/2024.04.25.591185; this version posted October 2, 2024. The copyright holder for this preprint (which was not certified by peer review) is the author/funder, who has granted bioRxiv a license to display the preprint in perpetuity. It is made available under aCC-BY 4.0 International license.

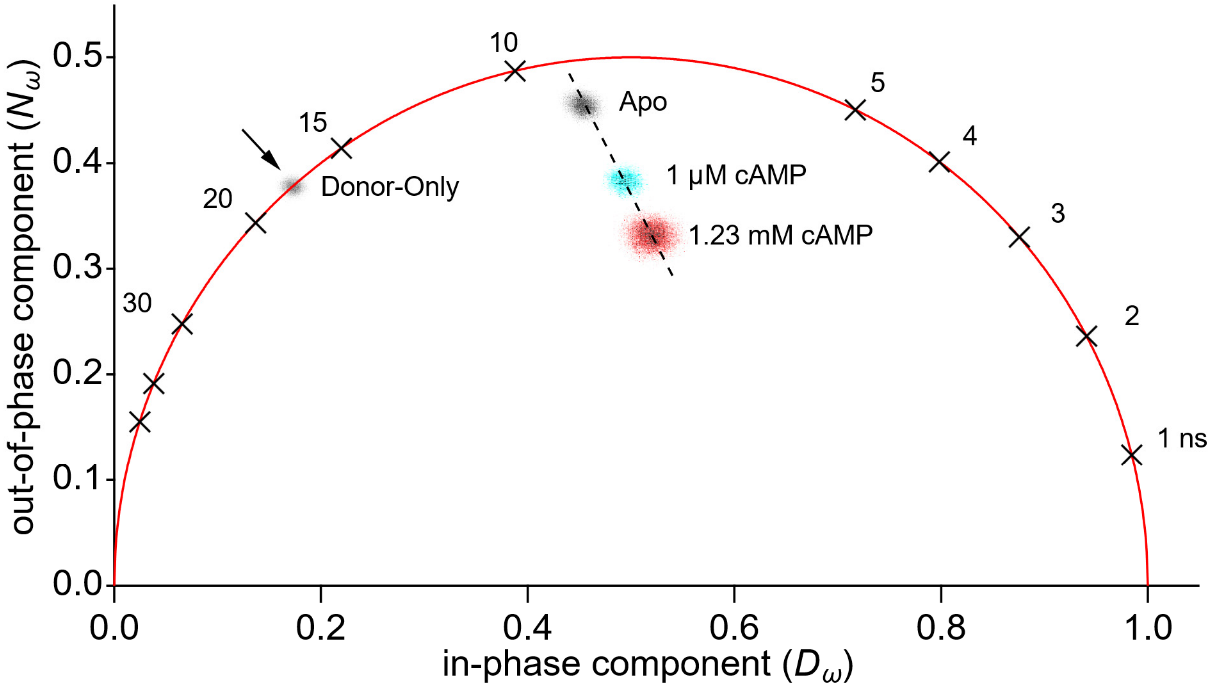

Fig. S4. Representative phasor plot of measured lifetimes of SthK$_{C\text{-term}}$-S361Acd-V416C alone (donor-only, grey), in the presence of $[Ru(bpy)_2phenM]^{2+}$ (apo, black), in the presence of 1 µM cAMP (cyan) and presence of 1.23 mM cAMP (red).

bioRxiv preprint doi: https://doi.org/10.1101/2024.04.25.591185; this version posted October 2, 2024. The copyright holder for this preprint (which was not certified by peer review) is the author/funder, who has granted bioRxiv a license to display the preprint in perpetuity. It is made available under aCC-BY 4.0 International license.

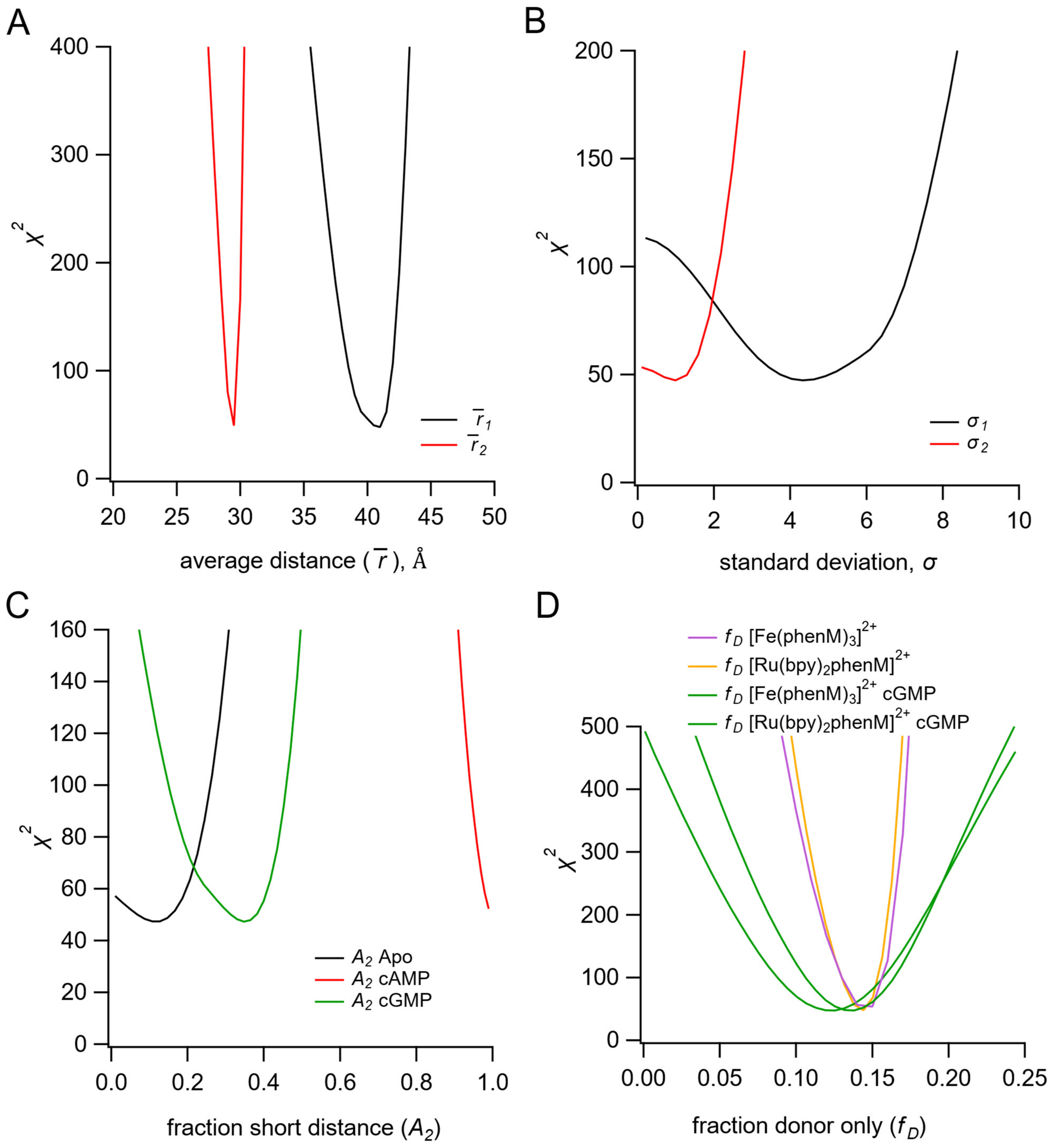

Fig. S5. Identifiability of parameters in the sum of two Gaussian distributions model, with global fitting $Fe(phenM)_3]^{2+}$ and $[Ru(bpy)_2phenM]^{2+}$ data. **(A-B)** Minimization of χ^2 values for the Gaussian average distance ($\bar{r}$) (A) and standard deviation (σ) (B) for the resting (black) and active states (red). **(C)** Minimization of χ^2 values for parameter A_2 for the conditions of apo, cAMP and cGMP. **(D)** Minimization of χ^2 values for the fraction of donor-only component (f_D) in protein samples for each experiment of apo and cAMP for $[Fe(phenM)_3]^{2+}$, of apo and cAMP for $[Ru(bpy)_2phenM]^{2+}$, of cGMP for $[Fe(phenM)_3]^{2+}$, and of cGMP for $[Ru(bpy)_2phenM]^{2+}$.
